# mRNA cap regulation in mammalian cell function and fate^☆^

**DOI:** 10.1016/j.bbagrm.2018.09.011

**Published:** 2019-03

**Authors:** Alison Galloway, Victoria H. Cowling

**Affiliations:** Centre for Gene Regulation and Expression, School of Life Sciences, University of Dundee, Dow Street, Dundee DD1 5EH, UK

## Abstract

In this review we explore the regulation of mRNA cap formation and its impact on mammalian cells. The mRNA cap is a highly methylated modification of the 5′ end of RNA pol II-transcribed RNA. It protects RNA from degradation, recruits complexes involved in RNA processing, export and translation initiation, and marks cellular mRNA as “self” to avoid recognition by the innate immune system. The mRNA cap can be viewed as a unique mark which selects RNA pol II transcripts for specific processing and translation. Over recent years, examples of regulation of mRNA cap formation have emerged, induced by oncogenes, developmental pathways and during the cell cycle. These signalling pathways regulate the rate and extent of mRNA cap formation, resulting in changes in gene expression, cell physiology and cell function.

## Highlights

• The mRNA cap is a methylated modification of the 5′ terminus of mRNA.
• RNA processing and translation factors are recruited to the mRNA cap.
• The mRNA cap protects transcripts from degradation and defines mRNA as “self”.
• Formation of the mRNA cap is regulated by cellular signalling pathways.
• mRNA cap regulation results in changes in gene expression and cell function.

## 1 The mRNA cap

RNA pol II-transcribed RNA typically constitutes less than 5% of the total RNA in mammalian cells. RNA pol II-transcribed RNA includes pre-mRNA (messenger RNA), pre-miRNA (micro RNA), pre-lncRNA (long non-coding RNA), snoRNAs (small nucleolar RNAs), and snRNA (small nuclear RNA). The majority of cellular RNA is rRNA (ribosomal RNA) and tRNA (transfer RNA) which is transcribed by RNA pol I and III. RNA pol II products, therefore, represent a functionally distinct group that must be processed, transported and, in the case of mRNA, translated, separately from other RNA species: this requires a mark of their identity. RNA pol II products are uniquely marked during transcription by the addition of a methylated guanosine cap structure to the 5′ terminus. The mRNA cap blocks 5′-3′ exonuclease-mediated degradation and recruits specific RNA processing, export and translation factors [1,2]. Removal of the cap (decapping) initiates degradation of mRNA [3]. Thus the cap is mechanistically involved in every stage of the mRNA lifecycle. Other RNA pol II-transcribed RNA species are also capped, but the cap structure and function varies. For example, pre-miRNA loses its cap during maturation, and snRNA and snoRNA caps can be further modified to a tri-methylguanosine (TMG) cap [4]. In this review we will focus on the regulation and role of the mRNA cap.

## 2 mRNA cap modifications

**Fig. 1:**
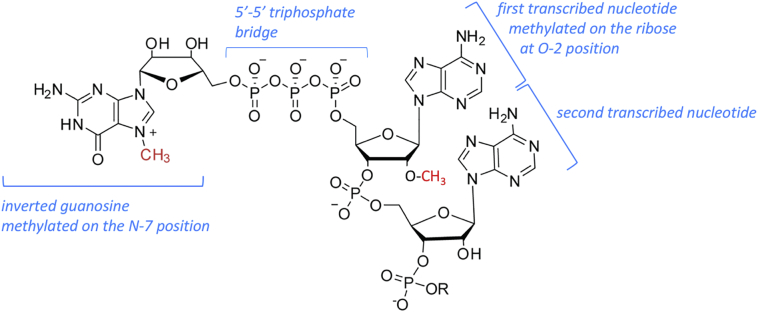
The mRNA cap. A predominant cap structure in mammalian cells is depicted. 7-Methylguanosine is linked to the first transcribed nucleotide *via* a 5′ to 5′ triphosphate bridge. The first transcribed nucleotide is methylated on the O-2 position of the ribose. Other methylations are also observed including first transcribed nucleotide adenosine methylation on position 6 and second transcribed nucleotide ribose O-2 methylation.

In mammals, the predominant cap structure is 7-methylguanosine linked *via* a 5′ to 5′ triphosphate bridge to the first transcribed nucleotide, which is methylated on the ribose O-2 position (denoted m7G(5′)ppp(5′)Xm, X is the first transcribed nucleotide) [2,5] Fig. 1. m7G(5′)ppp(5′)Xm was initially presumed to be present on all mRNA, however, due to advances in biochemistry, organ-specific and cell-specific levels of N-7 cap guanosine methylation and O-2 first nucleotide ribose methylation have been observed [[6], [7], [8], [9], [10]]. This suggests differential regulation of mRNA cap formation in different cell lineages and/or in response to specific signalling pathways. In addition, 2nd transcribed nucleotide ribose O-2 methylation and first nucleotide Adenosine N-6 methylation are also readily observed [11,12]. First nucleotide Adenosine N-6 methylation is an abundant modification, with m7G(5′)ppp(5′)m6Am contributing 20–30% of m7G(5′)ppp(5′)Xm mRNA caps in HeLa cells [13]. Since modifications such as Adenosine N-6 methylation are nucleotide-specific, and since cap binding proteins may have nucleotide preferences, the transcription start site impacts on the cap structure by determining the first transcribed nucleotide [14]. The range of modifications which are detected on internal positions of RNAs may also be present on the cap guanosine and cap proximal nucleotides. However, the enzymes that have been demonstrated to methylate the cap and adjacent nucleotides are specific for the cap structure and it is unlikely that enzymes which modify internal residues will also modify the cap [1,4].

Recently, a different type of cap structure, the nicotinamide adenine dinucleotide (NAD) cap, which was originally identified in bacteria and yeast, was identified in mammalian cells [8,15]. NAD caps targets transcripts for degradation [8]. Although the proportion of mRNA carrying a NAD cap is low in HEK293T cells, it may well be elevated and/or regulated in other cell lines or primary tissues [16]. The level of NAD capping is determined at least in part by the cellular concentration of NAD. The challenge going forward will be to detect lower abundance modifications and determine their functional significance. Determining the function of cap modifications in cells is greatly facilitated by identifying the enzymes which catalyse synthesis or removal.

## 3 Function of the mRNA cap

**Fig. 2:**
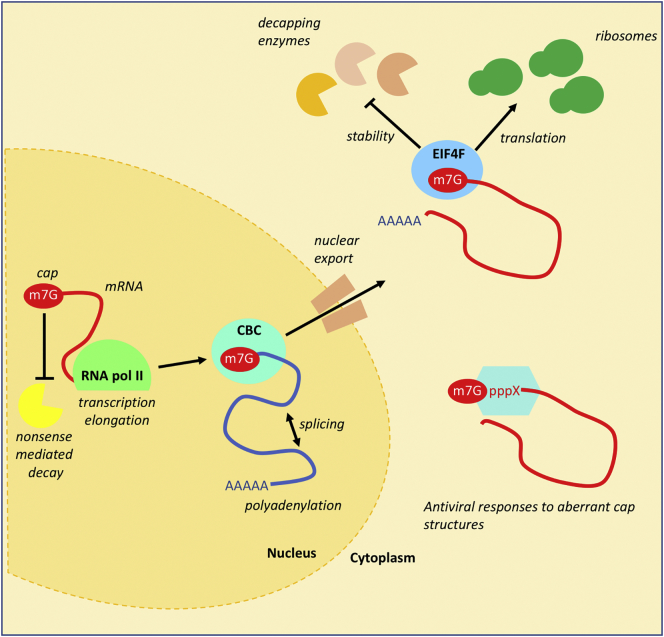
mRNA cap function. mRNA cap formation initiates during transcription. The mRNA cap protects pre-mRNA from decay during transcription. CBC (cap binding complex) binds to the mRNA cap and recruits proteins which mediate splicing, polyadenylation and export into the cytoplasm. eIF4F (eukaryotic initiation factor 4F) binds to the cap and recruits the 40S ribosomal subunit, initiating translation. The mature mRNA cap inhibits the action of 5′-3′ exonucleases until it is removed by the decapping enzymes. Antiviral responses can be induced by incomplete caps, particularly those with tri-phosphate ends or lacking O-2 methylation on the first and second transcribed nucleotides.

The known functions of the mRNA cap are mediated by its interactions with binding proteins and complexes (Fig. 2). A cap modification may influence the affinity of the cap to its various co-factors [[17], [18], [19]].

### 3.1 RNA processing and translation: CBC and eIF4F

CBC (cap binding complex) and eIF4F (eukaryotic initiation factor 4F) are the major mRNA cap binding complexes in mammalian cell lines. The predominantly nuclear CBC consists of a cap binding protein, NCBP2 (nuclear cap binding complex 2/CBP20) and its interacting partner, NCBP1 (nuclear cap binding complex 1/CBP80). CBC binds to the cap and recruits mechanistic proteins to the pre-mRNA, promoting splicing of the first intron, 3′-end processing, nuclear export and initiation of the pioneer round of translation [1,20]. For some mRNA, particularly histones, CBC can also be involved in subsequent rounds of translation [21]. For the majority of mRNA in the cytoplasm, the mRNA cap binds to the cap binding complex, eIF4F, which promotes translation initiation [4]. The eIF4F protein complex consists of a cap binding protein, eIF4E, a scaffold protein eIF4G and a helicase eIF4A (or their isoforms). When bound to the cap, eIF4F recruits the initiation factor eIF3, which in turn recruits the initiator tRNA and 40S ribosome subunit. eIF4E also has nuclear functions including in RNA export of specific transcripts [22]. The cap binding component of eIF4F, eIF4E, is regulated through the mTOR (mammalian target of rapamycin) and MNK1/2 (MAP Kinase Interacting Serine/Threonine Kinase) signalling pathways, coupling cap-dependent translation initiation to nutrient, oxygen and growth factor availability [23]. In contrast to eIF4F-mediated translation, CBC-mediated translation is resistant to mTOR-dependent inhibition [21,24].

Both CBC and eIF4F have a strong preference for the cap guanosine to be methylated. eIF4E binds to m7G(5′)ppp(5′)G with 1000-fold greater affinity than G(5′)ppp(5′)G, and NCBP2 has more than 150-fold greater affinity for m7G(5′)ppp(5′)G than G(5′)ppp(5′)G [25,26]. Both CBC and eIF4F also have increased affinity for the cap when the first transcribed nucleotide is a purine and a moderate increases in affinity for cap analogues when the first transcribed nucleotide is O-2 methylated. Consistent with this, first transcribed nucleotide O-2 methylation has a role in translation initiation [[27], [28], [29], [30]].

### 3.2 RNA processing and translation: alternative cap binding complexes

Homologues and alternatives of CBC and eIF4F introduce diversity into cap-dependent mRNA regulation. The most abundant CBC is NCBP1-NCBP2, however, NCBP3 is a m7G cap-binding protein which forms an alternative cap binding complex with NCBP1 [31]. In cells growing under steady-state conditions the NCBP1-NCBP2 and NCBP1-NCBP3 complexes functionally overlap, however, NCBP3 has a critical role in clearing viral infections. NCBP3 bears no homology to NCBP2 and in a separate study it was shown to have low affinity for m7GTP, leading the authors to conclude that it does not have a major role in cap binding [32]. However, NCBP3 may have higher affinity for complete cap structures, in which the first few transcribed nucleotides may be important.

eIF4E has two homologues eIF4E2 and eIF4E3 [33]. Although originally described as suppressive competitors of eIF4E with lower affinity for the cap structure, both have now been shown to regulate translation under particular conditions [34]. eIF4E2 has been shown to mediate translation in hypoxic conditions when eIF4E is inhibited. Hypoxia stimulates formation of a complex containing eIF4E2, HIF-2α (hypoxia inducible factor 2α), and RBM4 (RNA binding motif protein 4), which binds to RNAs containing RNA hypoxia response elements (rHRE) and recruits them to the ribosome. Certain mRNA bearing rHRE (hypoxia response elements) have enhanced dependency on eIF4E2 for translation.

eIF4E3 competes with eIF4E for cap binding when it is overexpressed and has been shown to decrease expression of oncogenic proteins and impair cell transformation [35]. When cells are treated with an MNK inhibitor, eIF4E3 expression is increased and mediates translation initiation [36]. The eIF4E3-dependent translatome overlaps with that of eIF4E, but certain mRNAs, including those involved in the NFκB pathway, are preferentially translated by eIF4E. eIF4E2 binds to the mRNA cap through a similar mechanism to eIF4E whereas eIF4E3 has an atypical binding mechanism [35,37]. Although eIF4E2 and eIF4E3 bind to m7G, the cap binding specificities of these alternative subunits have not been extensively investigated.

eIF3D, a subunit of the eIF3 complex, has cap binding activity which allows translation of particular mRNAs, including *cJUN* mRNA, in an eIF4E-independent mechanism [38]. eIF3D-mRNA binding is competitively inhibited by m7GDP and not GDP indicating a specificity towards N-7 methylated cap guanosine.

In addition to the two major cap binding complexes, CBC and eIF4F, newly identified cap binding proteins are expanding our understanding of cap function. LARP1 binds to the cap and the 5′ terminal oligo pyrimidine (TOP) motif present on transcripts encoding ribosomal proteins and selected translation factors [39]. By binding to the cap LARP1 stabilises transcripts [40], but blocks eIF4E binding and translation [41]. mTOR-dependent phosphorylation of LARP1 releases the mRNA cap allowing translation to occur, linking the production of ribosomes to cell growth [41]. Although LARP1 binds to a TOP motif with a m7G(5′)ppp(5′)C cap and not a decapped equivalent, it is not clear whether methylation of the cap structure contributes to binding.

For other cap binding proteins and complexes, the biological consequences of their cap binding activity is less well understood; Pumilio 2 has been shown to be capable of competing with eIF4E to inhibit translation [42], and the exon-junction complex core heterodimer Y14/Magoh binds to the mRNA cap and inhibits the decapping activity of DCP2 [43]. It is not yet known whether these proteins have specificity for particular RNAs.

### 3.3 Decapping and RNA decay

A major mechanism through which cap binding proteins enhance mRNA stability is by blocking the access of RNA decapping and decay enzymes [3]. The majority of decapping enzymes hydrolyse the triphosphate bridge connecting the inverted guanosine to the first transcribed nucleotide, removing either m7GMP or m7GDP from the 5′ end of the mRNA. DXO (Decapping Exoribonuclease) can also cleave between the first and second transcribed nucleotide [44]. Removal of the cap allows 5′-3′ decay to proceed.

There are several mammalian decapping enzymes which have different target preferences [3]. For example, the DCP2 is recruited to specific mRNA by RNA binding proteins and miRNAs (micro RNAs) that bind to cis acting elements on the transcript, whereas NUDT16 is more ubiquitous [3,45]. NUDT3 influences cell migration by through selective decapping of integrin β6 and lipocalin-2 mRNAs [46]. Several decapping enzymes also appear to have RNA binding activity which may aid target selection. For some decapping enzymes, variation in the cap structure itself influences decapping activity directly. Recently first nucleotide O-2 methylation was shown to prevent mRNA decapping by the enzyme DXO which has reduced affinity for caps with methylated first transcribed nucleotides [47]. DCP2 decapping activity is not affected by first transcribed nucleotide O-2 methylation, but is inhibited by N6 methylation of adenine in the first position. Thus, transcripts beginning with m6Am are resistant to miRNA mediated degradation [48]. N6A methylation is reversed by FTO (fat mass and obesity-associated protein), thereby influencing decapping and mRNA stability [48]. Humans with FTO loss-of-function mutations exhibit growth retardation and other issues, which may in part be due to aberrant cap metabolism and subsequent gene dysregulation [49].

Decapping prepares mRNA for 5′-3′ decay, however, the polyA tail is also susceptible to 3′-5′ degradation. PolyA tail shortening reduces the translation efficiency of mRNA and enhances decapping. Interestingly PARN (Poly-A specific ribonuclease), the enzyme responsible for polyA tail shortening is also a cap binding protein and PARN-mediated degradation of RNA ending in triphosphate is inefficient indicating that the cap marks mRNA for this specific degradation pathway [50].

### 3.4 Cap binding proteins involved in the antiviral response

The innate immune system is able to recognise non-self RNAs through pattern recognition receptors (PRRs) [51,52]. One such PRR, RIG-1 (retinoic acid inducible 1; DDX58, Dead Box Helicase 58) is able to recognise double stranded RNA with either cap structures lacking first transcribed nucleotide O-2 methylation, or with 5′ di or tri-phosphate structures [53,54]. O-2 methylation of the first transcribed nucleotide is most effective at blocking RIG-1 binding to m7G capped RNA, but second transcribed nucleotide O-2 methylation also has an impact. RIG-1 binding to non-self RNA stimulates an Interferon β response and increased expression of IFIT (IFN-induced protein with tetratricopeptide repeats) proteins, some of which mediate the innate immune response. Inhibition of O-2 methylation on endogenous mRNA can also induce these responses, which could mediate a stress response in certain cellular contexts [53]. IFIT proteins bind to single stranded RNA with aberrant cap structures and repress translation. Their binding specificities vary; IFIT1 binds to capped RNA with m7G(5′)ppp(5′)G, G(5′)ppp(5′)G or A(5′)ppp(5′)G caps with little selection, but cannot bind to RNA with first or second transcribed nucleotide ribose O2 methylated [55]. IFIT5 binds only to uncapped triphosphate ended RNAs [56]. IFIT proteins have a role in inhibiting translation of viral RNA, but it is not known whether they have a physiological role in repressing cellular RNAs with incomplete cap structures.

## 4 mRNA capping enzymes

**Fig. 3:**
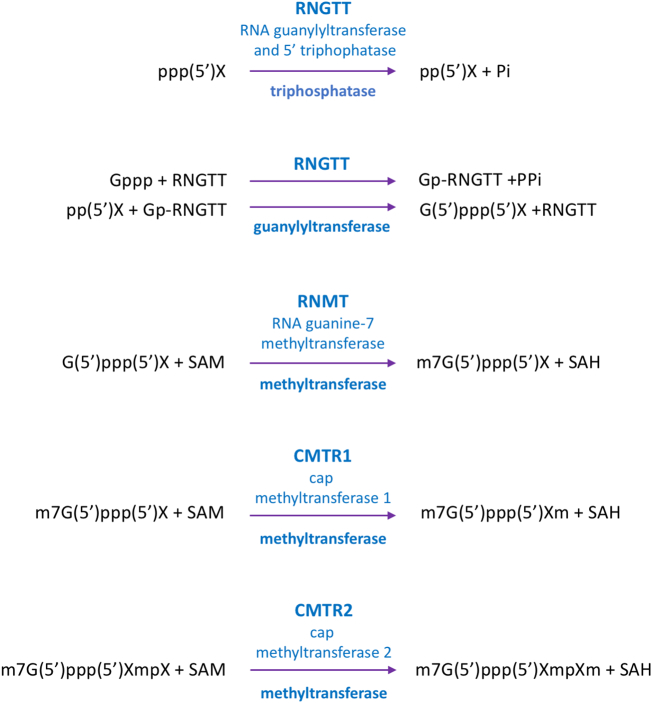
mRNA cap synthesis. The major capping reactions in mammalian cells are depicted. Other methylation events on first and second transcribed nucleotide are observed. Enzyme symbol, name and function in blue. SAM is s-adenosyl methionine. SAH is S-adenosyl homocysteine. RNMT, CMTR1, CMTR2 can all act on G(5′)ppp(5′)XpX; *i.e.* no prior methylation of the cap is required.

mRNA cap formation is catalysed by a series of enzymes [1,4]. The biochemical mechanism of mRNA cap formation is likely to be the same in all species in which it is found, Fig. 3. However the enzymes involved have diverged in different species [1,57]. Additionally, the mRNA capping enzymes have evolved to receive regulatory signals which can result in changes in their expression, localisation and activity. In particular, in vertebrates there are additional mRNA capping enzymes, regulatory domains and subunits that are absent in yeast, through which signalling pathways can regulate mRNA cap formation and therefore gene expression. In this review, we will discuss mechanisms by which the mammalian capping enzymes are regulated and the resultant impact on gene regulation, cell function and cell fate.

### 4.1 RNGTT

The first transcribed nucleotide of RNA pol II products retains the 5′ triphosphate, ppp(5′)X, to which the 7-methylguanosine cap is added, Fig. 3. Initially RNGTT (RNA guanylyltransferase and 5′ phosphatase; CE, Capping Enzyme), catalyses guanosine cap addition [58,59]. In vertebrates, RNGTT has two catalytic sites, a triphosphatase and guanylyltransferase. The triphosphatase removes the terminal phosphate to create diphosphate-terminated RNA, pp(5′)X. Subsequently the guanylyltransferase hydrolyses GTP (guanosine triphosphate) and forms a covalent linkage with the product, GMP, which is transferred to pp(5′)X to create the first cap structure G(5′)ppp(5′)X. RNGTT is recruited to Serine-5 phosphorylated C-terminal domain (CTD) of RNA pol II at the initiation of transcription and the enzyme is positioned to act on the nascent transcript immediately as it emerges from the polymerase [60]. Interaction with Ser-5 phosphorylated CTD also increases the activity of the guanylyltransferase [61]. Although guanylylation of the nascent transcript is reversible *in vitro*, in cells the reverse reaction is likely to be limited due to the interaction of RNGTT with Ser-5 phosphorylated CTD being restricted to the initial stages of transcription [62]. In other eukaryotes the triphosphatase and guanylyltransferase are distinct enzymes; having both activities on a single polypeptide allows efficient coupling of the reactions to create the guanosine cap.

### 4.2 RNMT

Addition of the inverted guanosine group to nascent transcripts creates the substrate cap for a series of methyltransferases [63]. RNMT (RNA guanine-7 methyltransferase), methylates the N-7 position of the guanosine cap [64,65]. The catalytic region of RNA guanine-7 methyltransferases is well conserved in different species, however the non-catalytic, N-terminal domain is divergent even between mammalian species [66]. A significant concentration of post-translational modifications (including acetylation, methylation, phosphorylation) are found on the RNMT N-terminal domain [67]. This domain is a substrate for modification by signalling pathways which regulate RNMT activity (discussed later) [68]. In human RNMT, the N-terminal domain restrains methyltransferase activity; its removal increases catalytic activity 2-fold [69]. Since the structure of this domain has not been determined, the mechanism by which it influences catalytic activity is unclear. The N-terminal domain is also required for efficient recruitment to chromatin [69]. RNMT is recruited to transcription initiation sites in a phospho-RNA pol II CTD-dependent manner, although direct interaction is unlikely.

### 4.3 RAM

In vertebrates, the cap guanosine N-7 methyltransferase, RNMT, has an activating subunit, RAM (RNMT-activating miniprotein) [70]. RAM stabilises the structure and positioning of the RNMT lobe and adjacent helix hinge, resulting in optimal positioning of helix A which contacts substrates in the active site [71]. RAM increases the binding of the methyl donor, s-adenosyl methionine (SAM), to the RNMT active site and may be particularly important for cap guanosine methylation under conditions of limiting SAM. RAM also has an RNA binding domain, which is not required for methyltransferase activity *in vitro*, whereas in cells it may be required to increase the efficiency of RNA substrate recruitment, or enhance the recruitment of specific substrates [72]. To date a consensus sequence for RAM binding has not been reported.

Although mRNA cap methylation occurs during the early stages of transcription in the nucleus, RNGTT and RNMT-RAM are also present in the cytoplasm where they can catalyse mRNA cap guanosine addition and methylation [73,74]. This mechanism may cap mRNA that escaped cap formation during transcription or decapped mRNA. In addition to its role in mRNA cap methylation, RNMT-RAM promotes transcription, independently of its role in mRNA cap methylation [75]. Suppression of RNMT-RAM expression results in over 80% loss in RNA pol II peaks on chromatin and a 2-fold drop in global transcription. RNMT-RAM interacts with nascent transcripts along their entire length and with transcription-associated factors including RNA pol II subunits, SPT4, SPT6 and PAFc. The emerging model is that interactions between RNMT-RAM, RNA and RNA pol II factors stimulate transcription [75].

### 4.4 CMTR1

In mammals, the ribose of the first and second transcribed nucleotides is methylated on the O-2 position by CMTR1 (Cap Methyltransferase 1) and CMTR2 (Cap methyltransferase 2), respectively [11,76]. CMTR1 is a multi-domain protein consisting of a G-patch domain, a RrmJ/FtsJ methyltransferase domain, a non-functional cap guanylyltransferase-like domain and a WW domain [27,77]. Deletion of the domains C-terminal to the methyltransferase, the guanylyltransferase-like and WW domains, reduces the activity of CMTR1 *in vitro* and therefore these domains may contribute to substrate recruitment and/or structural configuration. These CMTR1 domains may provide a platform by which co-factors and post-translational modifications can mediate regulation of ribose O-2 methylation. The CMTR1 WW domain interacts with Ser-5 phosphorylated C-terminal domain (CTD) of RNA Pol II [77,78]. Since CMTR1 methylates G(5′)ppp(5′)X and m7G(5′)ppp(5′)X equivalently, it is possible that CMTR1 acts prior to RNMT [76,79].

### 4.5 CMTR2

CMTR2, the second transcribed nucleotide ribose O-2 methyltransferase, also has several functional domains [11,79]. The N-terminal half of CMTR2 contains the methyltransferase domain. The C-terminus contains a methyltransferase-like domain, which does not have a competent active site but is required for CMTR2 methyltransferase activity. CMTR2-dependent methylation does not require a N-7 methylated cap guanosine or a first nucleotide O-2 methylated ribose, however these structures increase the efficiency of 2nd nucleotide ribose O-2 methylation [11]. CMTR2 is found in both the nucleus and cytoplasm of MCF7 cells [11].

### 4.6 Other capping enzymes

Enzymes involved in other cap methylations have not been identified to date [48]. As previously discussed, the enzymes which catalyse cap modifications are unlikely to be the same as the enzymes which catalyse internal RNA modifications since the cap structure fits into mRNA cap methyltransferase active sites [71,79]. The first transcribed nucleotide N6A methyltransferase is of particular interest since the m7G(5′)ppp(5′)m6Am cap is highly abundant in HeLa cells [13].

## 5 Major cellular mechanisms of mRNA cap regulation in mammals

The mRNA capping enzymes, similar to most enzymes can be regulated at several levels; expression, activity, recruitment to substrate (including subcellular localisation), and specificity for substrate. The majority of the signalling pathways which influence the capping enzymes deposit regulatory post-translational modifications on the enzymes or regulate the action or expression of co-factors. Since methyltransferases can be inhibited by their bi-product, s-adenosyl homocysteine (SAH), mRNA cap methylation can also be regulated by factors that regulate the enzyme SAHH (s-adenosyl homocysteine hydrolase) which hydrolyses SAH [80].

### 5.1 Serine-5 phosphorylated RNA pol II CTD

**Fig. 4:**
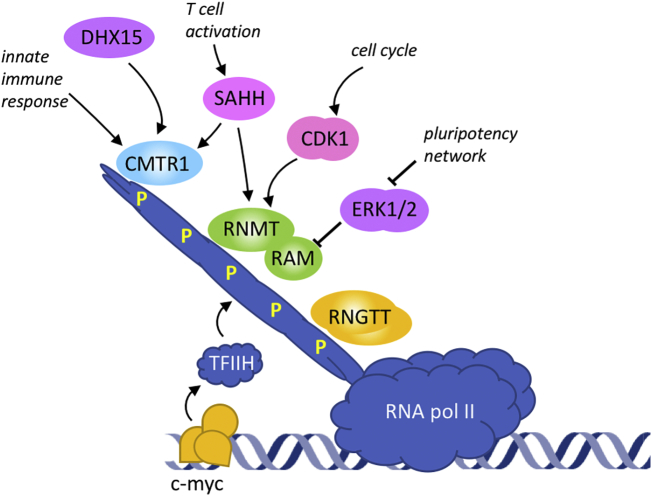
Regulation of mRNA cap synthesis. Phospho-RNA pol II CTD (C-terminal domain) recruits RNGTT and activates guanylyltransferase activity, and recruits RNMT and CMTR1. c-Myc, E2F-1 and other transcription factors promote RNA pol II phosphorylation. In embryonic stern cells, the pluripotency-associated gene network represses ERK 1/2 activity; during differentiation loss of pluripotency is associated with increased ERK1/2 activity which phosphorylates RAM-S36 resulting in ubiquitin-dependent degradation. T cell activation resulting in upregulation of SAHH expression which hydrolyses SAH, the inhibitory product of methylation reactions. CDK1-cyclin B1 phosphorylates RNMT T77 which increases methyltransferase activity. During the innate immune response CMTR1 is upregulated.

mRNA cap formation initiates during the early stages of transcription, with RNGTT recruitment to the RNA pol II large subunit, Fig. 4 [60,81]. As described earlier, RNGTT is recruited to Ser-5 phosphorylated RNA Pol II CTD, and the guanylyltransferase activity is stimulated by this interaction [61]. CMTR1 is also recruited to Ser-5 phospho-CTD and RNMT is recruited to RNA pol II, probably indirectly, in a phospho-CTD-dependent manner [69,77,78]. Currently, there is no evidence that RNMT and CMTR1 are activated by interaction with the RNA pol II CTD. Since Ser-5 phosphorylation of the RNA pol II CTD is a basic requirement of transcription, this recruitment and activation of the capping enzymes can be perceived as a basal mechanism of gene expression. However, many signalling pathways can impact on RNA pol II CTD phosphorylation, including transcription factors which increase CTD phosphorylation at specific genes [82]. c-Myc and E2F1 have been demonstrated to increase RNA pol II CTD phosphorylation and the proportion of their target transcripts with an m7G(5′)ppp(5′)X cap [7,[83], [84], [85]]. Furthermore, many transcription factors influence CTD phosphorylation, and they too may increase gene-specific cap formation.

### 5.2 Cell cycle

The N-terminus of RNMT is a non-catalytic regulatory domain which receives a heavy concentration of post-translational modification [67,69]. RNMT is phosphorylated on Thr-77 during late G2 phase, throughout mitosis and into early G1-phase [68], Fig. 4. Thr-77 phosphorylation increases RNMT activity, therefore providing a boost of cap guanosine N-7 methylation during early G1 phase, when the majority of transcription is occurring. In HeLa cells, CDK1-cyclin B is the predominant RNMT Thr-77 kinase. However the amino acid sequence surrounding Thr-77, Gly-Lys-Asp-Thr-Pro-Ser-Lys, indicates that many other proline-directed kinases may phosphorylate this site in other cells and/or in other cellular contexts.

### 5.3 Embryonic stem cell differentiation

The RNMT co-factor RAM activates RNMT methyltransferase activity and has an RNA binding domain which may enhance cap guanosine N-7 methylation *in vivo* [70,72]. When it was discovered, RAM was presumed to be a constitutive RNMT co-factor. In the cancer cell lines in which they were investigated, RNMT and RAM were isolated as heterodimers; monomers were unstable and undetectable. However when investigated in primary cells and *in vivo*, RNMT and RAM were found to be expressed differentially in different tissues, suggesting that these proteins can be stabilised by other co-factors or post-translational modifications in different cell types [86].

In embryonic stem (ES) cells, expression of RNMT and RAM is relatively high. However, the co-dependency of RNMT and RAM expression is minimal in ES cells and therefore their cellular concentrations can be independently modified. RAM expression is controlled by ERK1/2-dependent Ser-36 phosphorylation which triggers ubiquitin-dependent degradation. When cells are pluripotent, ERK1/2 activity is suppressed and RAM expression is maintained. During neural differentiation, ERK1/2 activity is upregulated resulting in RAM Ser-36 phosphorylation and ubiquitin-dependent degradation, whereas RNMT expression is largely maintained [86]. This regulation of RAM has both global and gene-specific impacts. In ES cells, RAM is required for the efficient translation of all mRNA, but is also required for the mRNA expression of pluripotency-associated genes. Repression of RAM during neural differentiation is required for repression of pluripotency-associated mRNAs and the emergence of neural markers. As with RNMT Thr-77, RAM Ser-36 lies within a motif, Pro-Pro-Glu-Ser-Pro-Pro, which indicates that it could be phosphorylated by other kinases in other cells or under other conditions. RAM expression is also repressed relative to RNMT in many other organs, indicating that its repression may be required for the differentiation of many other lineages. Of note RAM is upregulated with respect to RNMT in cardiac tissue, suggesting that it can have a role independent of cap guanosine methylation.

### 5.4 DHX15

The predominant CMTR1-interacting protein is DHX15, an RNA helicase with roles in RNA processing and ribosome biogenesis [78,87]. Approximately half of CMTR1 in HeLa cells is bound to DHX15. The DHX15 OB-fold binds to the CMTR1 G-patch domain, an interaction which represses methyltransferase activity by approximately 50%. Furthermore, when bound to DHX15, CMTR1 does not interact with RNA pol II, further restricting its activity. These mechanisms may constrain O-2 methylation to a predominantly co-transcriptional event. Conversely, CMTR1 activates DHX15 helicase activity which has a wide range of roles in RNA processing [87].

### 5.5 Innate immunity

CMTR1 was first characterised as KIA0082/ISG95, a protein implicated in the response to interferon treatment and viral infection [[88], [89], [90], [91]]. CMTR1-dependent first transcribed nucleotide ribose O-2 methylation is important for the identification of mRNA as “self”, preventing its recognition by innate immune response proteins [53,54,91]. CMTR1 expression is increased following interferon treatment which is likely to protect cellular RNAs. Upregulation of CMTR1 may also impact cellular mRNA translation during the interferon response.

### 5.6 Cancer

Some of the factors which stimulate mRNA cap formation are elevated in certain tumour types, including expression of c-Myc, E2F1 and CDK1, and RNA pol II phosphorylation. Overexpression of RNMT enhances cellular transformation alone as well as in combination with MYC and RAS, and inhibition of RNGTT specifically targets cells with high levels of c-Myc expression, raising the possibility of the mRNA capping enzymes as therapeutic targets [85,92]. Furthermore, the elevated rates of transcription observed in cells expressing oncogenes may render them particularly dependent on high rates of mRNA cap formation. Since methyltransferases are inhibited by their bi-product, SAH, mRNA cap methylation can be regulated by changes in expression of SAHH (S-adenosyl homocysteine hydrolase), the enzyme which hydrolyses SAH [80]. SAHH is a c-Myc-induced gene and has been demonstrated to be required for mRNA cap formation, protein synthesis and cell proliferation following c-Myc deregulation [80]. SAHH is also upregulated following T cell activation which may facilitate the increased requirement for mRNA cap methylation and other methylation reactions during increased gene expression [80]. The mRNA cap methyltransferases may be more appropriate than RNGTT as therapeutic targets since their active-site inhibitors tend to be of low polarity and able to cross the plasma membrane. In the case of RNMT, targeting RAM may retain basal cap methyltransferase activity, reducing toxicity [70,71]. Furthermore, since cancer cell lines have enhanced dependency on RAM compared to non-transformed cells, targeting RAM may enhance selectivity for transformed cells [70,86].

Cap binding proteins including LARP1 and eIF4E also have oncogenic activities, probably *via* the control of key target genes [93,94]. The mTOR and MNK pathways that regulate eIF4E-dependent translation are currently being targeted individually and together to antagonise mRNA translation in cancer cells [95]. Inhibitors which directly disrupt the interaction between eIF4E and eIF4G have also been developed [96]. Presumably, inhibitors of the capping enzymes will have overlapping sets of target transcripts with eIF4E inhibitors. There is also evidence that certain decapping enzymes may be useful therapeutic targets [97].

The viral dsRNA sensor RIG-1 is associated with tumour suppressive functions which are being investigated as targets in cancer immunotherapy [[98], [99], [100]]. RNAs with 5′ tri-phosphate activate RIG-1, including bifunctional 5′ tri-phosphate siRNAs that silence oncogenic mRNAs [[101], [102], [103], [104], [105], [106]]. These RNAs have anti-tumour effects in a variety of cancer cell lines and mouse tumour engraftment models. Mechanisms for RIG-1 agonist-mediated tumour suppression included the induction of apoptosis through a variety of effectors and the activation of immune responses from dendritic cells and CD8 T lymphocytes. Since knockdown of CMTR1 has proven effective in stimulating RIG-1, presumably by increasing endogenous mRNAs lacking first transcribed nucleotide O-2 methylation, CMTR1 inhibitors may also have anti-tumour activities [53]. RIG-1 also has ligand-independent roles, notably in restraining proliferation during granulopoiesis [107] and leukaemias [108,109], and in mediating the effects of IFNα (interferon alpha) [108]. The potential contribution of self-RNAs to regulate RIG-1 activity in these contexts has not been thoroughly explored.

## 6 Challenges of mRNA cap research

Early during mRNA cap discovery, three structures were identified and defined, Cap 0 (G(5′)ppp(5′)X), cap 1 (m7G(5′)ppp(5′)Xm), and cap 2 m7G(5′)ppp(5′)XmXm (both first and second transcribed nucleotides methylated on the ribose O-2 position) [99]. We now recognise the existence of NAD caps and additional modifications of the first transcribed nucleotides are emerging. Furthermore we identify G(5′)ppp(5′)X, G(5′)ppp(5′)Xm, G(5′) and m7G(5′)ppp(5′)X in significant quantities in liver extracts (unpublished data). Following the identification of novel, low abundance cap modifications, the challenge is it to determine their function. Some mRNA cap-dependent processes (*e.g.* translation initiation) can be readily reconstituted *in vitro*. However other mRNA cap-dependent processes (*e.g.* splicing, export) are most reliably studied in intact cells because they require intact cellular components and/or are most efficient when coupled to other cellular processes. Therefore the investigation of the function of novel modifications is enhanced by the identification of the enzymes involved and their deletion/modification in cells. It is worth noting that not all modifications of the first few transcribed nucleotides will have a function (or not on every transcript). Non-deleterious modifications may have no selective pressure to be removed.

Another major challenge in mRNA cap research is to determine how many transcribed nucleotides are constituents of the cap. In trypanosomes and other kinetoplastids, the cap is added to a 39 nucleotide “splice leader” transcript [110,111]. The process of “trans-splicing” adds this splice leader to mRNAs, which are transcribed from polycistrons. In this splice leader the first four transcribed nucleotides are methylated to create the cap 4 structure, m^7^G(5′)ppp(5′)m^6^_2_AmpAmpCmpm^3^Um [[112], [113], [114]]. Repression of cap methylation in kinetoplastids results in reduced trans-splicing and translation [[112], [113], [114], [115]]. Since we now may identify modifications on the 3rd and 4th transcribed nucleotides in mammals, should these all be considered as part of the cap structure? We propose that the cap structure should be described on a functional basis, and therefore the question will become, which transcribed nucleotides influence interaction with the different cap binding complexes? Of note, the cap binding complex, CBC, is a two subunit, 100 kDa complex in mammals whereas it is a 5 subunit, 300 kDa complex in trypanosomes which may utilise additional contacts with the extended cap 4 structure [116].

Here we have described how mRNA cap modifications are synthesised, how the activity of the enzymes involved is regulated and how they influence mRNA processing and gene expression. Future studies will likely identify novel cap structure, characterise the regulation of cap formation *in vivo* and uncover the role of cap diversity in the control of mammalian cell behaviour.

## Transparency document

Transparency document. Image 1
